# (4-Chloro-2-{[(pyridin-2-ylmeth­yl)imino]­meth­yl}phenolato)iodido(methanol)zinc(II)

**DOI:** 10.1107/S160053681100417X

**Published:** 2011-02-09

**Authors:** Hong-Wei Huang

**Affiliations:** a College of Chemistry and Biology Engineering, Yichun University, Yichun 336000, People’s Republic of China

## Abstract

The title Schiff base zinc(II) complex, [Zn(C_13_H_10_ClN_2_O)I(CH_3_OH)], was synthesized by the reaction of 5-chloro­salicyl­aldehyde, 2-amino­methyl­pyridine and zinc iodide in methanol. The Zn^II^ atom is five-coordinated by one phenolate O atom, one imine and one pyridine N atom of the Schiff base ligand, one methanol O atom and one I atom, forming a distorted square-pyramidal geometry, with the I atom at the apical site. The dihedral angle between the benzene and pyridine rings is 22.9 (2)°. In the crystal, centrosymmetrically related mol­ecules are linked through inter­molecular O—H⋯O hydrogen bonds, forming dimers.

## Related literature

For the structures of Schiff bases and their complexes, see: Ali *et al.* (2008 ▶); Eltayeb *et al.* (2007 ▶); Datta *et al.* (2009 ▶); Zhao *et al.* (2010 ▶); Temel *et al.* (2010 ▶); Naveenkumar *et al.* (2010 ▶).
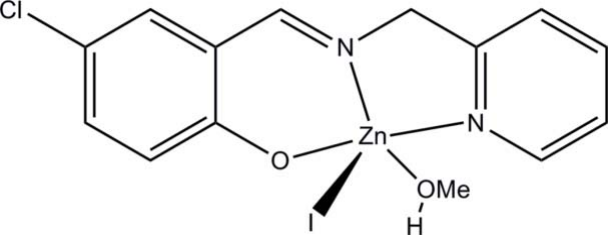


## Experimental

### 

#### Crystal data

[Zn(C_13_H_10_ClN_2_O)I(CH_4_O)]
*M* *_r_* = 469.99
Monoclinic, 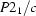

*a* = 7.0769 (9) Å
*b* = 12.7212 (16) Å
*c* = 18.225 (2) Å
β = 98.994 (1)°
*V* = 1620.5 (3) Å^3^
*Z* = 4
Mo *K*α radiation
μ = 3.59 mm^−1^
*T* = 298 K
0.20 × 0.20 × 0.18 mm

#### Data collection

Bruker SMART CCD area-detector diffractometer
Absorption correction: multi-scan (*SADABS*; Sheldrick, 1996 ▶) *T*_min_ = 0.534, *T*_max_ = 0.564
9273 measured reflections
3522 independent reflections
2947 reflections with *I* > 2σ(*I*)
*R*_int_ = 0.021

#### Refinement

*R*[*F* ^2^ > 2σ(*F* ^2^)] = 0.025
*wR*(*F* ^2^) = 0.058
*S* = 1.04
3522 reflections
195 parameters
1 restraint
H atoms treated by a mixture of independent and constrained refinement
Δρ_max_ = 0.37 e Å^−3^
Δρ_min_ = −0.91 e Å^−3^

### 

Data collection: *SMART* (Bruker, 1998 ▶); cell refinement: *SAINT* (Bruker, 1998 ▶); data reduction: *SAINT*; program(s) used to solve structure: *SHELXS97* (Sheldrick, 2008 ▶); program(s) used to refine structure: *SHELXL97* (Sheldrick, 2008 ▶); molecular graphics: *SHELXTL* (Sheldrick, 2008 ▶); software used to prepare material for publication: *SHELXTL*.

## Supplementary Material

Crystal structure: contains datablocks global, I. DOI: 10.1107/S160053681100417X/rz2553sup1.cif

Structure factors: contains datablocks I. DOI: 10.1107/S160053681100417X/rz2553Isup2.hkl

Additional supplementary materials:  crystallographic information; 3D view; checkCIF report

## Figures and Tables

**Table 1:** Hydrogen-bond geometry (Å, °)

*D*—H⋯*A*	*D*—H	H⋯*A*	*D*⋯*A*	*D*—H⋯*A*
O2—H2⋯O1^i^	0.86 (3)	1.79 (3)	2.643 (3)	176 (3)

Symmetry code: (i) 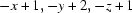.

**Fig. 1.**
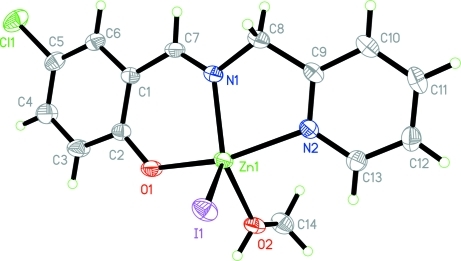
The molecular structure of the title compound, with 30% displacements ellipsoids.

**Fig. 2.**
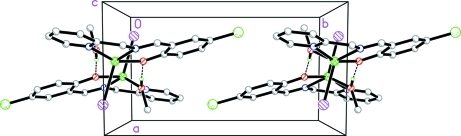
The molecular packing of the title compound, viewed along the c axis. Hydrogen atoms not involved in hydrogen bonds (dashed lines) are omitted for clarity.

## supplementary crystallographic information

### Comment

Schiff bases and their complexes have attracted much attention for their interesting structures (Ali *et al.*, 2008; Eltayeb *et al.*, 2007; Datta *et al.*, 2009; Zhao *et al.*, 2010; Temel *et al.*, 2010; Naveenkumar *et al.*, 2010). In this paper, the title new Schiff base zinc(II) complex, Fig. 1, is reported.

The Zn atom in the complex is five-coordinated by one phenolate O atom, one imine and one pyridine N atoms of the Schiff base ligand, one methanol O atom, and one iodide atom to form a distorted square pyramidal geometry. The dihedral angle between the benzene and the pyridine rings is 22.9 (2)°. In the crystal structure (Fig. 2), centrosymmetrically related molecules are linked through intermolecular O—H···N hydrogen bonds (Table 1) to form dimers.

### Experimental

Equimolar quantities (0.1 mmol each) of 5-chlorosalicylaldehyde, 2-aminomethylpyridine, and zinc iodide were mixed and stirred in methanol for 30 min at reflux. After keeping the filtrate in air for a few days, colourless block crystals suitable for X-ray analysis were formed.

### Refinement

H2 attached to O2 was located from a difference Fourier map, and refined with the O–H distance restrained to 0.85 (1) Å, and with *U*_iso_ restrained to 0.08 Å^2^. The remaining H atoms were placed in calculated positions and constrained to ride on their parent atoms, with C—H distances of 0.93–0.97 Å, and with *U*_iso_(H) = 1.2*U*_eq_(C) or 1.5*U*_eq_(C) for methyl H atoms.

### Crystal data

**Table:**

[Zn(C_13_H_10_ClN_2_O)I(CH_4_O)]	*F*(000) = 912
*M**_r_* = 469.99	*D*_x_ = 1.926 Mg m^−^^3^
Monoclinic, *P*2_1_/*c*	Mo *K*α radiation, λ = 0.71073 Å
Hall symbol: -P 2ybc	Cell parameters from 3746 reflections
*a* = 7.0769 (9) Å	θ = 2.7–27.8°
*b* = 12.7212 (16) Å	µ = 3.59 mm^−^^1^
*c* = 18.225 (2) Å	*T* = 298 K
β = 98.994 (1)°	Block, colorless
*V* = 1620.5 (3) Å^3^	0.20 × 0.20 × 0.18 mm
*Z* = 4	

### Data collection

**Table:**

Bruker SMART CCD area-detector diffractometer	3522 independent reflections
Radiation source: fine-focus sealed tube	2947 reflections with *I* > 2σ(*I*)
graphite	*R*_int_ = 0.021
ω scans	θ_max_ = 27.0°, θ_min_ = 2.0°
Absorption correction: multi-scan (*SADABS*; Sheldrick, 1996)	*h* = −9→8
*T*_min_ = 0.534, *T*_max_ = 0.564	*k* = −16→15
9273 measured reflections	*l* = −23→17

### Refinement

**Table:**

Refinement on *F*^2^	Primary atom site location: structure-invariant direct methods
Least-squares matrix: full	Secondary atom site location: difference Fourier map
*R*[*F*^2^ > 2σ(*F*^2^)] = 0.025	Hydrogen site location: inferred from neighbouring sites
*wR*(*F*^2^) = 0.058	H atoms treated by a mixture of independent and constrained refinement
*S* = 1.04	*w* = 1/[σ^2^(*F*_o_^2^) + (0.0266*P*)^2^ + 0.3654*P*] where *P* = (*F*_o_^2^ + 2*F*_c_^2^)/3
3522 reflections	(Δ/σ)_max_ = 0.003
195 parameters	Δρ_max_ = 0.37 e Å^−^^3^
1 restraint	Δρ_min_ = −0.91 e Å^−^^3^

### Special details

**Table:**

Geometry. All e.s.d.'s (except the e.s.d. in the dihedral angle between two l.s. planes) are estimated using the full covariance matrix. The cell e.s.d.'s are taken into account individually in the estimation of e.s.d.'s in distances, angles and torsion angles; correlations between e.s.d.'s in cell parameters are only used when they are defined by crystal symmetry. An approximate (isotropic) treatment of cell e.s.d.'s is used for estimating e.s.d.'s involving l.s. planes.
Refinement. Refinement of *F*^2^ against ALL reflections. The weighted *R*-factor *wR* and goodness of fit *S* are based on *F*^2^, conventional *R*-factors *R* are based on *F*, with *F* set to zero for negative *F*^2^. The threshold expression of *F*^2^ > σ(*F*^2^) is used only for calculating *R*-factors(gt) *etc*. and is not relevant to the choice of reflections for refinement. *R*-factors based on *F*^2^ are statistically about twice as large as those based on *F*, and *R*- factors based on ALL data will be even larger.

### Fractional atomic coordinates and isotropic or equivalent isotropic displacement parameters (Å^2^)

**Table:**

	*x*	*y*	*z*	*U*_iso_*/*U*_eq_	
Zn1	0.56550 (5)	1.00055 (2)	0.370141 (16)	0.03411 (9)	
Cl1	0.80941 (15)	0.43991 (6)	0.42899 (5)	0.0673 (3)	
I1	0.20664 (3)	1.054046 (16)	0.337917 (10)	0.04454 (7)	
O1	0.5675 (3)	0.87992 (14)	0.44157 (10)	0.0428 (5)	
O2	0.6619 (3)	1.10027 (17)	0.45789 (11)	0.0461 (5)	
N1	0.6673 (3)	0.89862 (17)	0.29675 (11)	0.0343 (5)	
N2	0.6831 (3)	1.10583 (17)	0.29614 (12)	0.0353 (5)	
C1	0.6966 (4)	0.7424 (2)	0.37342 (14)	0.0331 (6)	
C2	0.6271 (4)	0.7827 (2)	0.43675 (14)	0.0350 (6)	
C3	0.6226 (5)	0.7138 (2)	0.49658 (16)	0.0502 (8)	
H3	0.5819	0.7388	0.5394	0.060*	
C4	0.6768 (5)	0.6104 (2)	0.49358 (17)	0.0517 (8)	
H4	0.6702	0.5664	0.5338	0.062*	
C5	0.7407 (5)	0.5715 (2)	0.43160 (17)	0.0443 (7)	
C6	0.7511 (4)	0.6356 (2)	0.37262 (16)	0.0394 (6)	
H6	0.7951	0.6086	0.3310	0.047*	
C7	0.7123 (4)	0.8024 (2)	0.30781 (15)	0.0357 (6)	
H7	0.7599	0.7676	0.2697	0.043*	
C8	0.6893 (5)	0.9462 (2)	0.22567 (15)	0.0450 (7)	
H8A	0.5762	0.9319	0.1896	0.054*	
H8B	0.7983	0.9150	0.2076	0.054*	
C9	0.7177 (4)	1.0624 (2)	0.23317 (15)	0.0364 (6)	
C10	0.7742 (4)	1.1230 (3)	0.17688 (15)	0.0451 (7)	
H10	0.7966	1.0917	0.1329	0.054*	
C11	0.7966 (4)	1.2294 (3)	0.18702 (17)	0.0492 (8)	
H11	0.8336	1.2712	0.1499	0.059*	
C12	0.7637 (4)	1.2735 (2)	0.25260 (17)	0.0480 (7)	
H12	0.7803	1.3452	0.2610	0.058*	
C13	0.7061 (4)	1.2099 (2)	0.30520 (17)	0.0437 (7)	
H13	0.6817	1.2400	0.3493	0.052*	
C14	0.8502 (5)	1.1310 (3)	0.48795 (17)	0.0548 (8)	
H14A	0.8972	1.0869	0.5296	0.082*	
H14B	0.8498	1.2029	0.5040	0.082*	
H14C	0.9315	1.1240	0.4507	0.082*	
H2	0.585 (4)	1.104 (2)	0.4897 (14)	0.055 (9)*	

### Atomic displacement parameters (Å^2^)

**Table:**

	*U*^11^	*U*^22^	*U*^33^	*U*^12^	*U*^13^	*U*^23^
Zn1	0.03959 (19)	0.03446 (17)	0.03043 (16)	0.00566 (13)	0.01222 (13)	0.00098 (12)
Cl1	0.0921 (7)	0.0349 (4)	0.0725 (6)	0.0165 (4)	0.0054 (5)	−0.0032 (4)
I1	0.03836 (12)	0.05586 (13)	0.04069 (12)	0.01147 (9)	0.01019 (8)	0.00744 (8)
O1	0.0617 (13)	0.0352 (10)	0.0351 (10)	0.0147 (9)	0.0191 (9)	0.0028 (8)
O2	0.0553 (14)	0.0511 (12)	0.0363 (11)	−0.0032 (10)	0.0210 (10)	−0.0105 (9)
N1	0.0387 (13)	0.0371 (12)	0.0285 (11)	0.0009 (10)	0.0102 (9)	−0.0023 (9)
N2	0.0354 (13)	0.0385 (12)	0.0333 (12)	0.0014 (10)	0.0096 (10)	0.0029 (10)
C1	0.0311 (14)	0.0365 (14)	0.0320 (13)	0.0031 (11)	0.0055 (11)	−0.0022 (11)
C2	0.0359 (15)	0.0360 (14)	0.0327 (14)	0.0056 (11)	0.0045 (11)	−0.0002 (11)
C3	0.072 (2)	0.0460 (17)	0.0355 (15)	0.0140 (16)	0.0157 (15)	0.0035 (13)
C4	0.072 (2)	0.0411 (16)	0.0431 (17)	0.0130 (16)	0.0112 (15)	0.0108 (14)
C5	0.0494 (18)	0.0337 (14)	0.0481 (17)	0.0080 (13)	0.0023 (14)	−0.0022 (12)
C6	0.0381 (16)	0.0379 (15)	0.0421 (16)	0.0038 (12)	0.0063 (12)	−0.0065 (12)
C7	0.0356 (15)	0.0401 (15)	0.0330 (14)	0.0003 (12)	0.0104 (11)	−0.0102 (12)
C8	0.061 (2)	0.0469 (17)	0.0299 (14)	0.0002 (14)	0.0149 (14)	−0.0022 (12)
C9	0.0304 (15)	0.0473 (16)	0.0323 (14)	0.0017 (12)	0.0079 (11)	0.0051 (12)
C10	0.0418 (17)	0.061 (2)	0.0338 (15)	0.0001 (14)	0.0104 (13)	0.0063 (13)
C11	0.0449 (18)	0.0580 (19)	0.0461 (17)	−0.0019 (14)	0.0111 (14)	0.0198 (15)
C12	0.0471 (18)	0.0418 (16)	0.0557 (19)	0.0003 (14)	0.0101 (15)	0.0103 (14)
C13	0.0485 (18)	0.0404 (16)	0.0433 (16)	0.0046 (13)	0.0108 (13)	0.0030 (13)
C14	0.057 (2)	0.066 (2)	0.0415 (17)	−0.0016 (17)	0.0095 (15)	0.0005 (15)

### Geometric parameters (Å, °)

**Table:**

Zn1—O1	2.0111 (18)	C4—C5	1.373 (4)
Zn1—N1	2.071 (2)	C4—H4	0.9300
Zn1—O2	2.071 (2)	C5—C6	1.361 (4)
Zn1—N2	2.158 (2)	C6—H6	0.9300
Zn1—I1	2.6060 (5)	C7—H7	0.9300
Cl1—C5	1.746 (3)	C8—C9	1.496 (4)
O1—C2	1.314 (3)	C8—H8A	0.9700
O2—C14	1.415 (4)	C8—H8B	0.9700
O2—H2	0.86 (3)	C9—C10	1.391 (4)
N1—C7	1.272 (3)	C10—C11	1.372 (4)
N1—C8	1.460 (3)	C10—H10	0.9300
N2—C9	1.330 (3)	C11—C12	1.372 (4)
N2—C13	1.340 (3)	C11—H11	0.9300
C1—C6	1.412 (4)	C12—C13	1.365 (4)
C1—C2	1.419 (3)	C12—H12	0.9300
C1—C7	1.438 (4)	C13—H13	0.9300
C2—C3	1.403 (4)	C14—H14A	0.9600
C3—C4	1.373 (4)	C14—H14B	0.9600
C3—H3	0.9300	C14—H14C	0.9600
			
O1—Zn1—N1	88.42 (8)	C4—C5—Cl1	119.8 (2)
O1—Zn1—O2	89.96 (8)	C5—C6—C1	121.3 (3)
N1—Zn1—O2	140.91 (9)	C5—C6—H6	119.4
O1—Zn1—N2	156.05 (8)	C1—C6—H6	119.4
N1—Zn1—N2	77.17 (8)	N1—C7—C1	126.4 (2)
O2—Zn1—N2	89.42 (8)	N1—C7—H7	116.8
O1—Zn1—I1	104.65 (6)	C1—C7—H7	116.8
N1—Zn1—I1	116.29 (6)	N1—C8—C9	111.1 (2)
O2—Zn1—I1	101.87 (6)	N1—C8—H8A	109.4
N2—Zn1—I1	98.90 (6)	C9—C8—H8A	109.4
C2—O1—Zn1	130.13 (16)	N1—C8—H8B	109.4
C14—O2—Zn1	130.14 (18)	C9—C8—H8B	109.4
C14—O2—H2	112 (2)	H8A—C8—H8B	108.0
Zn1—O2—H2	113 (2)	N2—C9—C10	121.3 (3)
C7—N1—C8	118.7 (2)	N2—C9—C8	116.7 (2)
C7—N1—Zn1	127.11 (18)	C10—C9—C8	122.0 (2)
C8—N1—Zn1	114.18 (16)	C11—C10—C9	119.2 (3)
C9—N2—C13	118.8 (2)	C11—C10—H10	120.4
C9—N2—Zn1	115.00 (17)	C9—C10—H10	120.4
C13—N2—Zn1	125.93 (18)	C12—C11—C10	119.2 (3)
C6—C1—C2	119.1 (2)	C12—C11—H11	120.4
C6—C1—C7	116.5 (2)	C10—C11—H11	120.4
C2—C1—C7	124.4 (2)	C13—C12—C11	118.7 (3)
O1—C2—C3	119.3 (2)	C13—C12—H12	120.7
O1—C2—C1	123.4 (2)	C11—C12—H12	120.7
C3—C2—C1	117.3 (2)	N2—C13—C12	122.9 (3)
C4—C3—C2	121.8 (3)	N2—C13—H13	118.6
C4—C3—H3	119.1	C12—C13—H13	118.6
C2—C3—H3	119.1	O2—C14—H14A	109.5
C5—C4—C3	120.5 (3)	O2—C14—H14B	109.5
C5—C4—H4	119.7	H14A—C14—H14B	109.5
C3—C4—H4	119.7	O2—C14—H14C	109.5
C6—C5—C4	120.0 (3)	H14A—C14—H14C	109.5
C6—C5—Cl1	120.2 (2)	H14B—C14—H14C	109.5

### Hydrogen-bond geometry (Å, °)

**Table:**

*D*—H···*A*	*D*—H	H···*A*	*D*···*A*	*D*—H···*A*
O2—H2···O1^i^	0.86 (3)	1.79 (3)	2.643 (3)	176 (3)

Symmetry codes: (i) −*x*+1, −*y*+2, −*z*+1.
